# Prevalence of and Factors Associated With High Blood Pressure Among Adolescents in India

**DOI:** 10.1001/jamanetworkopen.2022.39282

**Published:** 2022-10-31

**Authors:** Anil Vasudevan, Tinku Thomas, Anura Kurpad, Harshpal S. Sachdev

**Affiliations:** 1 Department of Pediatric Nephrology, St Johns Medical College and Hospital, St John’s Medical College, Bengaluru, India; 2 Department of Biostatistics, St John’s Medical College, Bengaluru, India; 3 Department of Physiology, St John’s Medical College, Bengaluru, India; 4 Paediatrics and Clinical Epidemiology, Sitaram Bhartia Institute of Science and Research, New Delhi, India

## Abstract

**Importance:**

High blood pressure (BP) in children and adolescents is becoming one of the most common health conditions worldwide and is much more widely prevalent than previously thought.

**Objective:**

To estimate the prevalence of high BP in adolescents in India and identify associated factors.

**Design, Setting, and Participants:**

This cross-sectional study is a secondary analysis of data from the Comprehensive National Nutrition Survey (CNNS, 2016-2018), which used a multistage, stratified, probability proportion to size cluster sampling design to enroll a nationally representative sample of households and individuals aged 10 to 19 years across all states and union territories of India. Adolescents with acute or chronic illness, physical deformity, mental illness, or cognitive disability were excluded from the survey. Because BP was measured only in children between 10 and 19 years of age, only data from children within this age group were included for secondary analyses. Data analysis was performed from March 2021 to April 2022.

**Exposures:**

Anthropometry.

**Main Outcomes and Measures:**

On the basis of the 2017 American Academy of Pediatrics guidelines, high BP was defined as stage 1 and 2 hypertension, with BP above the 95th percentile in children younger than 13 years and greater than 130/80 mm Hg in children 13 years or older. The association of age, sex, region, socioeconomic status, body mass index, fasting blood glucose, hemoglobin A_1c_, and lipid profile with high BP were examined using log binomial regression.

**Results:**

Among 16 182 eligible children aged 10 to 19 years (mean [SD] age, 14.2 [2.8] years; 7849 [48.5%] female and 8333 [51.5%] male), 11 718 had valid BP data with 3 repeated readings. the prevalence of high BP was 35.1% (95% CI, 31.5%-38.9%) in children aged 10 to 12 years and 25.1% (95% CI, 22.5%-28.0%) in children 13 years or older. Overweight and obesity were associated with a higher risk of high BP in both younger (prevalence ratio, 1.17; 95% CI, 1.04-1.34) and older children (prevalence ratio, 1.33; 95% CI, 1.18-1.49). The prevalence of high BP in younger children with stunting was high at 40.1% (95% CI, 31.9%-48.9%) and was 21.9% (95% CI, 18.2%-26.1%) among older children with stunting. In both age groups, high BP coexisted with other cardiovascular disease risk factors, such that adolescents with high fasting blood glucose, high hemoglobin A_1c_, high triglyceride, and high low-density lipoprotein cholesterol levels had a higher risk of high BP.

**Conclusions and Relevance:**

In this cross-sectional study, the prevalence of high BP, along with cardiovascular risk factors, was substantial in Indian adolescents. There is a need to screen and identify adolescents who have high BP and initiate interventions to control the burden of hypertension and its consequences in India.

## Key Points

**Question:**

Is high blood pressure (BP) common in adolescents in India, and what are the factors associated with high BP in this population?

**Findings:**

In this cross-sectional study of 11 718 children and adolescents aged 10 to 19 years from the Comprehensive National Nutrition Survey 2016-2018, the prevalence of high BP was 35.1% in children aged 10 to 12 years and 25.1% in those 13 years or older. Overweight and obesity along with cardiovascular disease risk factors, such as high fasting blood glucose, high triglyceride, and high low-density lipoprotein cholesterol levels, were associated with high BP.

**Meaning:**

These findings suggest that the prevalence of high BP in Indian adolescents is substantially high and coexists with other cardiovascular disease risk factors.

## Teaser

This cross-sectional study of adolescents in India examines the prevalence of high blood pressure and factors associated with high blood pressure in this population.

## Introduction

High blood pressure (BP) in children and adolescents is becoming one of the most common health conditions worldwide and is much more widely prevalent than previously thought.^1,2^ This condition during childhood frequently progresses to adult hypertension.^3,4^ Early detection and appropriate intervention for high BP and hypertension in childhood are therefore extremely important because these conditions are related to cardiovascular morbidity and mortality in adulthood and are commonly associated with diabetes, dyslipidemia, and obesity.^5,6,7,8^

High BP could also occur in those children who do not appear to be anthropometrically at risk. This occurrence is part of the extended double burden of malnutrition with a paradoxical co-occurrence of metabolic obesity in anthropometrically undernourished children, which was described in a 15-year-old data set from urban schools in Delhi, India,^9^ and recently confirmed in a nationally representative survey.^10^ Thus, whether high BP also occurs in anthropometrically undernourished Indian adolescents is important to know.

Although no nationally representative estimates are available of the prevalence of pediatric hypertension or high BP in India, some school-based studies have found a 2% to 20.5% prevalence of hypertension in children, especially in adolescents.^11^ However, for the first time in India, a national population-based quality-controlled survey is available, called the Comprehensive National Nutrition Survey (CNNS), conducted in 2016 to 2018.^12^ This survey recorded BP in a large number of adolescents (defined in this study as individuals aged 10-19 years) using standardized methods. Recent guidelines are also available for diagnosing high BP in children: updated clinical practice guidelines have been defined for diagnosing hypertension in children and adolescents in 2017, with several changes to age-dependent normative thresholds.^13^ The objectives of this study were to examine the prevalence of high BP in Indian adolescents in a national survey and to identify the associated factors, including noncommunicable disease risk factors, in this population.

## Methods

This cross-sectional study is a secondary analysis of the CNNS data, collected between 2016 and 2018 under the aegis of the Ministry of Health and Family Welfare, Government of India, in collaboration with UNICEF and the Population Council. Details of survey design and sampling methods are published elsewhere.^12^ Briefly, a multistage, stratified, probability proportion to size cluster sampling design was used to select a nationally representative sample of households and individuals aged 0 to 19 years across all 29 states of India. Households with individual(s) between 0 and 19 years of age were randomly selected from rural and urban primary sampling units; children and adolescent members were classified into 3 strata (0-4, 5-9, and 10-19 years of age). Ethical approval was obtained from the ethics committee of Post Graduate Institute of Medical Education and Research in Chandigarh, India. All data and samples were collected after written informed consent was obtained from parents or caregivers for children aged 10 to 17 years (as well as assent from those aged 11-17 years) and from adolescents aged 18 to 19 years. The reporting of the study followed the Strengthening the Reporting of Observational Studies in Epidemiology (STROBE) reporting guideline.^14^

### Measurement of BP

Blood pressure was measured using the same standardized protocol in all children. Automated devices were used to minimize observer bias or digit preferences. Trained medical laboratory technicians recorded 3 readings with an appropriately sized cuff in the right upper arm with the study participant in a sitting position with a gap of at least 2 minutes between the readings. All BP measurements that were 200 mm Hg or less were considered valid, and the rest (<0.05% of all BP readings) were not considered for this analysis. Valid second and third BP readings were considered to obtain the mean systolic BP (SBP) and diastolic BP (DBP). High BP was defined using the 2017 American Academy of Pediatrics guideline based on the normative distribution of BP in healthy children (eTable 1 in the Supplement).^13^ The guideline classified children as having normal BP, elevated BP, and stage 1 and stage 2 hypertension according to their age, sex, and Centers for Disease Control and Prevention growth chart based on height percentiles.^15^

### Anthropometry

Age- and sex-standardized height for age and body mass index (BMI; calculated as weight in kilograms divided by height in meters squared) for age *z* scores were calculated using the World Health Organization Growth Reference Standards.^16^ Children were classified as having growth stunting if the height for age *z* score was less than −2 SDs of the World Health Organization child growth standards median. Children were classified as having overweight or obesity if the BMI for age *z* score was greater than 1 and as underweight if the BMI for age *z* score was less than −2 SDs. All height for age and BMI for age *z* scores less than −6 and greater than 6 were excluded.

### Biochemical Parameters

Blood samples were collected in trace-element free tubes. Serum was separated, aliquoted, and stored frozen. Fasting blood glucose was measured by spectrophotometry using the hexokinase method, whereas hemoglobin A_1c_ (HbA_1c_) was estimated using high-performance liquid chromatography. Serum total cholesterol, low-density lipoprotein cholesterol (LDL-C), and high-density lipoprotein cholesterol (HDL-C) levels were measured by spectrophotometry using the cholesterol oxidase/esterase peroxidase, direct measure/cholesterol oxidase, and direct measure–polyethylene glycol/cholesterol oxidase methods, respectively. Serum triglyceride level was measured by the enzymatic end point method. Dysglycemia was considered when the fasting blood glucose level was 100 mg/dL or greater (to convert to millimoles per liter, multiply by 0.0555) or the HbA_1c_ value was 5.7% or more (to convert to a proportion of total hemoglobin, multiply by 0.01).^17^ An abnormal serum total cholesterol level was defined as 200 mg/dL or greater (to convert to millimoles per liter, multiply by 0.0259).^18^ We considered borderline abnormality cutoffs of 90 mg/dL or greater for high triglyceride levels (to convert to millimoles per liter, multiply by 0.0113), 110 mg/dL or greater for high LDL-C levels (to convert to millimoles per liter, multiply by 0.0259), and 45 mg/dL or less for low HDL-C (to convert to millimoles per liter, multiply by 0.0259) because the prevalence of definitely abnormal values was low (<5% for LDL-C) for evaluating meaningful associations.^18^

### Statistical Analysis

Sampling weights based on sampling probabilities of children at each stage of sampling for biological measurements were used for national and state level estimates to ensure that national and state representative estimates were obtained. All analyses were performed separately within 2 age categories, 10 to 12 years and 13 to 19 years, because the criteria for classification of BP differed between these age groups. The classification of BP was verified using the SAS macro (SAS Institute Inc) for assessment of BP percentiles, referred to in the guidelines.^13^ The national prevalences of elevated BP, stage 1 hypertension, and stage 2 hypertension (eTable 1 in the Supplement) were estimated with 95% CIs. Because no follow-up BP measurements were available for the confirmation of true hypertension status, the binary variable of high BP was used for further analysis with stage 1 and stage 2 groups combined to represent high BP and normal and elevated BP to form the reference (normal/elevated) group for all comparisons (eTable 1 in the Supplement). The difference in prevalence of high BP between the male and female children was observed graphically by nonparametric smoothed curves with 95% CIs. The association of sociodemographic and anthropometric characteristics with high BP was examined using log binomial regression with cluster robust estimation of the SE of coefficients. All factors associated with high BP along with area of residence (urban vs rural), sex, and age of child in years were considered in multiple regression models performed separately for those aged 10 to 12 years and 13 years or older to obtain covariate adjusted prevalence ratios (PRs). Unadjusted and adjusted PRs with 95% CIs are reported. The differential association of overweight or obesity with high BP in children with and without stunting was examined as an interaction in the regression model adjusted for the quintiles of household wealth index. The associations of cardiometabolic risk factors, such as high fasting blood glucose, HbA_1c_, total cholesterol, total triglycerides, LDL-C, and HDL-C levels, were explored in the 2 age groups using log binomial regression. The combined association of these factors along with overweight and obesity was considered as a count of multiple cardiometabolic risk factors (overweight and obesity, high fasting blood glucose level, high total cholesterol level, and high LDL-C level) present in each child and was also considered in a separate regression analysis to identify the role of clustering of multiple cardiometabolic risk factors on high BP. Sensitivity analyses were performed after selecting data with a less than 15–mm Hg difference between second and third systolic BP readings and a less than 10–mm Hg difference between second and third diastolic BP readings.

Data analysis was performed from March 2021 to April 2022. A 2-sided *P* < .05 was considered to be statistically significant for the log binomial regression analysis. All analyses were performed with Stata software, version 16.0 (StataCorp LLC).

## Results

### General Characteristics of Study Participants

**Figure 1.**
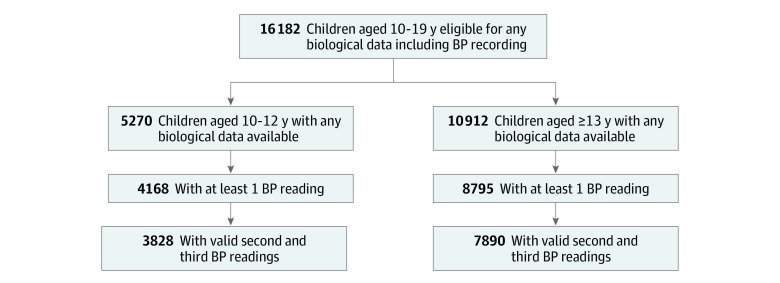
Flowchart of Sample Selection for the Analysis of High Blood Pressure (BP) by Age Group

A total of 16 182 children aged 10 to 19 years (mean [SD] age, 14.2 [2.8] years; 7849 [48.5%] female and 8333 [51.5%] male) had BP and blood biochemistry data available, and valid BP data with 3 repeated readings were available for 11 718 of these children (72.4%) (Figure 1). No statistically significant difference was found in sociodemographic characteristics between the sample of 10- to 19-year-old children in whom BP was measured and those in whom BP was not measured (eTable 2 in the Supplement). However, the prevalence of overweight was higher in the sample that had BP measurements performed (survey weighted estimates, 5.8% vs 2.9%; *P* = .001).

The mean (SD) age of the 11 718 participants in whom BP data were available was 14.0 (0.1) years, and 5649 (48.2%) were female. All further results are reported as survey weighted estimates for those aged 10 to 12 years (younger group) and those aged 13 to 19 years (older group). The prevalence of stunting was 24.8% in the younger age group and 27.0% in the older age group. The prevalence of underweight was 23.0% in the younger children and 17.0% in the older children. The prevalence of overweight was 5.8% and the prevalence of obesity was 1.5% in both age groups.

### Distribution of BP in Children

**Figure 2.**
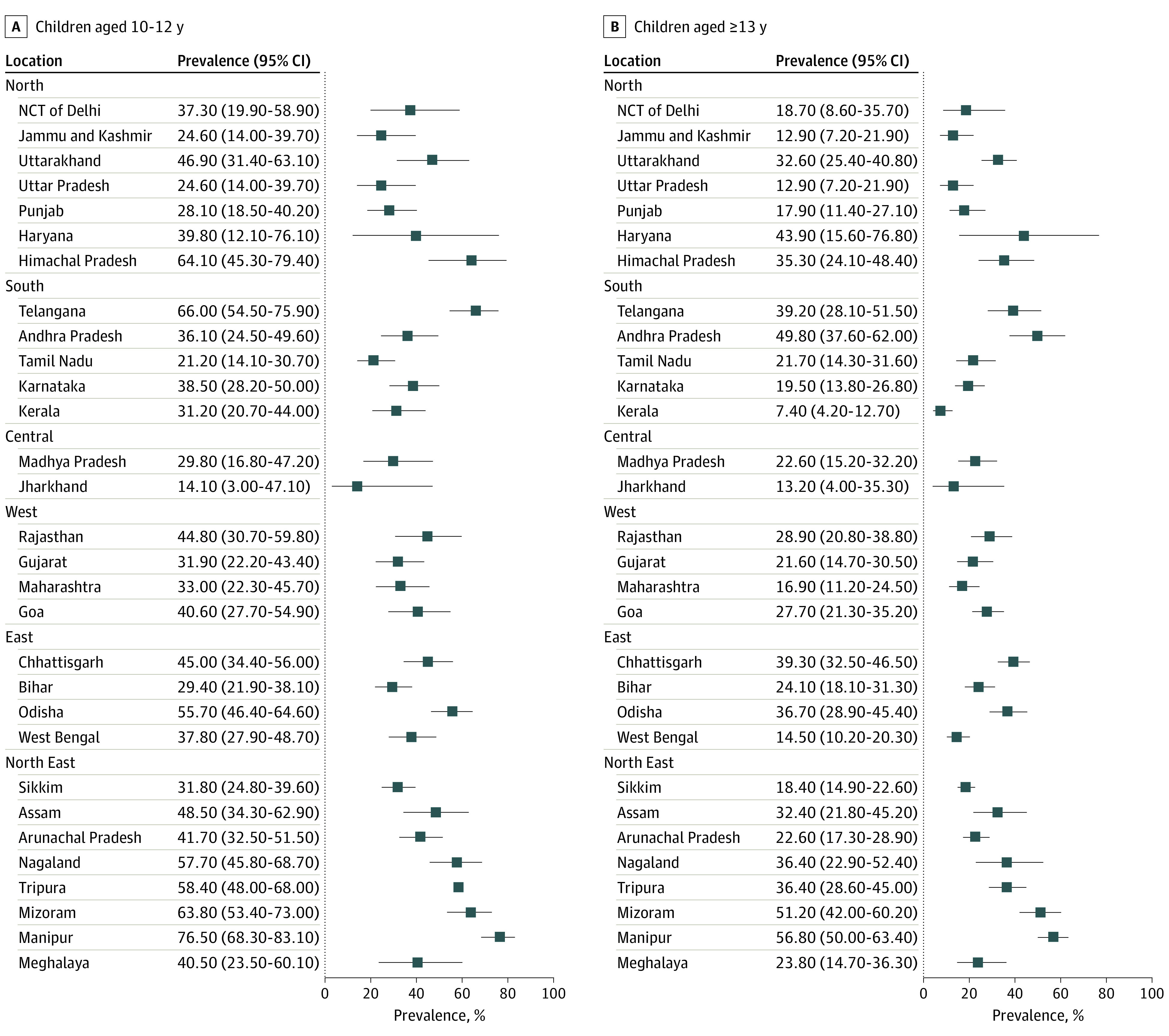
Statewide Prevalence of High Blood Pressure With 95% CIs NCT indicates National Capital Territory.

The mean SBP and DBP was higher by almost 2 mm Hg in older compared with younger children (Table 1). The SBP was lower in girls by 2.0 mm Hg (95% CI, 1.5-2.5 mm Hg) compared with boys in the older age group. However, DBP was comparable between both sexes and in both age groups (eFigure, A and B in the Supplement). The mean height percentile of the children, according to the Centers for Disease Control and Prevention criteria, was in the 17th percentile (95% CI, 16th-18th percentile). The national prevalence of high BP (stage 1 and 2 combined) was higher in the younger group at 35.1% (95% CI, 31.5%-38.9%) compared with 25.1% (95% CI, 22.5%-28.0%) in the older group (Table 1). The statewide prevalence of high BP is given in Figure 2A and B. There was no evidence of clustering of geographic regions with high prevalence.

**Table 1.** Distribution of Blood Pressure and Its Categories According to American Academy of Pediatrics Criteria^13^

Category	Mean (95% CI)	
	Age 10-12 y	Age ≥13 y
Blood pressure, mm Hg		
Systolic	109.3 (108.5-110.0)	112.6 (111.8-113.6)
Diastolic	71.9 (71.2-72.6)	73.8 (72.9-74.8)
Categories of blood pressure, %^a^		
Normal	48.9 (44.5-53.3)	64.1 (61.2-66.8)
Elevated blood pressure	15.9 (13.8-18.4)	10.8 (9.4-12.4)
Stage 1 hypertension	28.7 (25.3-32.4)	20.2 (18.1-22.6)
Stage 2 hypertension	6.4 (4.9-8.4)	4.9 (3.3-7.2)
High blood pressure (stage 1 and 2 hypertension)	35.1 (31.5-38.9)	25.1 (22.5-28.0)

^a^ Survey weighted estimates using national sampling weights.

### Prevalence of High BP by Sociodemographic and Anthropometric Risk Factors

The prevalence of high BP in rural areas was as high as that in urban areas, at 35.8% vs 33.4% in the younger group and approximately 25% in both areas in the older group (Table 2). The prevalence was also comparable between both sexes within each age group. In the younger group, children in the richest wealth category had a lower prevalence of high BP (PR, 0.82; 95% CI, 0.70-0.96) compared with children from the poorest wealth category. This difference was not observed in the older group. The prevalence of high BP was higher in children with overweight or obesity in both age groups. The increased prevalence was 17% in the younger group and 32% in the older group (PR, 1.32; 95% CI, 1.18-1.49). The sensitivity analyses performed after selecting data with less than a 15–mm Hg difference between the second and third SBP readings and less than a 10–mm Hg difference between second and third diastolic BP readings also gave similar results. When examined by specific BMI categories, the prevalence of high BP was highest in the group with obesity (eTable 3 in the Supplement).

**Table 2.** Prevalence of High BP by Sociodemographic and Other Individual Characteristics in Children Aged 10 to 12 Years

Characteristic	No. of children	Prevalence of high BP, % (95% CI)	PR (95% CI) of high BP	
			Unadjusted	Adjusted^a^
**Children aged 10-12 y **				
Area				
Rural	2176	35.8 (31.2-40.6)	1 [Reference]	1 [Reference]
Urban	1652	33.4 (28.4-38.7)	0.88 (0.80-0.96)	0.92 (0.83-1.01)
Sex				
Male	2012	33.2 (28.5-38.3)	1 [Reference]	1 [Reference]
Female	1816	37.2 (32.7-41.9)	1.04 (0.97-1.12)	1.04 (0.97-1.13)
Wealth index				
Poorest	317	38.4 (28.9-49.0)	1 [Reference]	1 [Reference]
Poor	517	33.1 (25.2-42.0)	0.98 (0.83-1.15)	0.96 (0.82-1.13)
Middle	780	35.4 (29.1-42.3)	1.01 (0.87-1.18)	1.00 (0.86-1.17)
Rich	1084	36.5 (29.7-43.8)	0.90 (0.78-1.04)	0.90 (0.78-1.05)
Richest	1130	32.2 (26.1-39.0)	0.83 (0.71-0.96)	0.83 (0.71-0.97)
BMI categories				
Underweight or normal	3424	34.3 (30.5-38.3)	1 [Reference]	1 [Reference]
Overweight or obesity	362	35.5 (25.9-46.4)	1.10 (0.97-1.25)	1.17 (1.04-1.34)
Stunting				
Yes	810	40.1 (31.9-48.9)	1.17 (1.08-1.28)	1.16 (1.06-1.26)
No	2984	33.6 (30.1-37.3)	1 [Reference]	1 [Reference]
**Adolescents aged ≥13 y **				
Area				
Rural	4420	25.1 (21.9-28.5)	1 [Reference]	1 [Reference]
Urban	3470	25.3 (20.6-30.6)	0.92 (0.84-1.00)	0.91 (0.83-1.01)
Sex				
Male	4057	25.9 (22.4-29.8)	1 [Reference]	1 [Reference]
Female	3833	24.3 (21.0-27.9)	1.00 (0.93-1.07)	1.00 (0.93-1.08)
Wealth index				
Poorest	522	22.4 (16.1-30.2)	1 [Reference]	1 [Reference]
Poor	1053	23.7 (18.7-29.6)	1.22 (1.02-1.47)	1.28 (1.05-1.55)
Middle	1638	23.7 (20.1-27.8)	1.26 (1.05-1.51)	1.30 (1.08-1.58)
Rich	2151	24.7 (21.0-28.9)	1.15 (0.96-1.38)	1.22 (1.01-1.48)
Richest	2526	30.1 (24.6-36.1)	1.12 (0.94-1.34)	1.20 (0.98-1.46)
BMI categories				
Underweight or normal	6530	24.1 (21.2-27.3)	1 [Reference]	1 [Reference]
Overweight or obesity	589	35 (28.6-41.9)	1.30 (1.15-1.46)	1.33 (1.18-1.49)
Stunted				
Yes	1861	21.9 (18.2-26.1)	0.92 (0.84-1.01)	0.92 (0.84-1.01)
No	5264	25.8 (22.5-29.3)	1 [Reference]	1 [Reference]

Abbreviations: BMI, body mass index (calculated as weight in kilograms divided by height in meters squared); BP, blood pressure; PR, prevalence ratio.

^a^ Prevalence ratios with 95% CIs adjusted for age, area, sex, wealth index, and BMI category.

With regard to double burden of malnutrition, the prevalence of high BP in children with stunting (height for age *z* score <−2) was high at 40.1% (95% CI, 31.9%-48.9%) compared with 33.6% (95% CI, 30.1%-37.3%) in children without stunting (height for age *z* score ≥−2). The association of overweight and obesity with high BP was further examined within stratified samples of children with and without stunting. The association of overweight and obesity was different by stunting status (eTable 4 in the Supplement). Among younger children, the association of overweight and obesity with high BP was higher in children with stunting (PR, 1.52; 95% CI, 1.18-1.96). In contrast, among older children, a similar association of high BP with overweight and obesity was observed in children with and without stunting. Overall, there was a synergistic effect of stunting and overweight toward high BP in younger children (PR, 1.34; *P* = .04 for interaction), after adjusting for all covariates. Wealth index alone was associated with high BP in the subgroups of children with stunting and children with overweight. However, all covariates considered in the overall model (sex of the child, area of residence, and wealth quintiles) were considered for all subgroup analyses. The prevalence of high BP was also high among children with underweight (BMI for age *z* score <−2) in both age groups (32.2% [95% CI, 27.1%-37.7%] for younger children and 21.5% [95% CI, 17.4%-26.1%] for older children). However, this prevalence was not statistically significantly higher than that observed in the normal weight children.

### High BP and Cardiometabolic Risk Factors

The prevalence of high BP in children with high fasting blood glucose levels was 46.4% compared with 35.0% in those with normal blood glucose levels among the younger children (Table 3). The adjusted PR of high BP in younger children with high blood glucose levels was 1.17 (95% CI, 1.06-1.29). In older children, the PR was 1.24 (95% CI, 1.12-1.35). High triglyceride and high LDL-C levels were also associated with high BP in both younger and older children. High HbA_1c_ levels were associated with high BP only in older children. High serum total cholesterol levels or low HDL-C levels were not independently associated with high BP in either age group. When the coexistence of multiple cardiometabolic risk factors (overweight or obesity, high fasting blood glucose level, and/or high total cholesterol level) was considered (eTable 5 in the Supplement), the PR of high BP was high for 1 or 2 additional cardiometabolic risk factors compared with none in younger children, and in older children, having 1 additional risk factor increased the risk of high BP. The most commonly occurring cardiometabolic risk factors were overweight or obesity and a high fasting blood glucose level in the older group.

**Table 3.** Coexistence of Cardiometabolic Risk Factors With High BP

Risk factor	Prevalence of high BP, % (95% CI)	PR (95% CI) of high BP	
		Unadjusted	Adjusted^a^
**Children aged 10-12 y**			
High fasting blood glucose (≥100 mg/dL)	46.4 (37.0-56.2)	1.18 (1.07-1.31)	1.17 (1.06-1.29)
High HbA_1c_ (≥5.7%)	34.4 (29.1-40.1)	1.08 (0.96-1.22)	NA
High total cholesterol (≥200 mg/dL)	36.7 (21.1-55.6)	1.13 (0.94-1.35)	NA
High triglycerides (≥90 mg/dL)	39.4 (33.8-45.4)	1.25 (1.15-1.36)	1.26 (1.16-1.37)
High LDL-C (≥110 mg/dL)	37.0 (29.6-45.1)	1.12 (1.01-1.26)	1.13 (1.01-1.26)
Low HDL-C (≤45 mg/dL)	32.3 (25.6-39.8)	0.98 (0.90-1.07)	NA
**Adolescents aged ≥13 y**			
High fasting blood glucose (≥100 mg/dL)	29.1 (21.0-38.8)	1.24 (1.13-1.37)	1.24 (1.12-1.35)
High HbA_1c_ (≥5.7%)	28.5 (23.2-34.4)	1.14 (1.02-1.27)	1.12 (1.00-1.25)
High total cholesterol (≥200 mg/dL)	36.5 (19.6-57.6)	1.14 (0.96-1.36)	NA
High triglycerides (≥90 mg/dL)	27.0 (23.0-31.5)	1.17 (1.08-1.27)	1.15 (1.06-1.25)
High LDL-C (≥110 mg/dL)	33.0 (24.4-42.8)	1.17 (1.05-1.30)	1.12 (1.00-1.25)
Low HDL-C (≤45 mg/dL)	23.1 (19.8-26.8)	0.95 (0.88-1.03)	NA

Abbreviations: BMI, body mass index (calculated as weight in kilograms divided by height in meters squared); BP, blood pressure; HbA_1c_, hemoglobin A_1c_; HDL-C, high-density lipoprotein cholesterol; LDL-C, low-density lipoprotein cholesterol; NA, not applicable; PR, prevalence ratio.

SI conversion factors: To convert glucose to millimoles per liter, multiply by 0.0555; HbA_1c_ to a proportion of total hemoglobin, multiply by 0.01; triglycerides to millimoles per liter, multiply by 0.0113; and cholesterol, LDL-C, and HDL-C to millimoles per liter, multiply by 0.0259.

^a^ Prevalence ratios with 95% CIs adjusted for age, area, sex, wealth index, and BMI category. The sample sizes for multiple regression analysis of different cardiometabolic risk factors ranged from 2960 to 3804 in children aged 10 to 12 years and 6151 to 7868 in children 13 years or older.

## Discussion

In this study, we report the cross-sectional prevalence and associations of high BP in a large, nationally representative survey of adolescents in India. Overall, the prevalence of high BP was substantial, occurring in one-third of the younger age group (aged 10-12 years) and one-fourth of the older age group (aged 13-19 years), which would amount to approximately 69 million adolescents with high BP in India based on 2011 census data. The prevalence of high BP noted in the CNNS in the current study is greater than that reported in most studies^19,20,21^ from India, although previous estimates were variable, ranging from 2.2% to 25.1%. In one of the largest cross-sectional studies that was conducted in central India, involving 11 312 children between 5 and 15 years of age (5305 girls and 6007 boys), high BP was found in 6.8% of the boys and 7.0% of the girls.^22^ In a meta-analysis^12^ of 25 published studies from India (total sample of 27 682 participants), the pooled estimate of prevalence of high BP was 7.6% (95% CI, 6.1%-9.1%). Despite significant heterogeneity among studies (*I*^2^ = 96.6%; *P* < .001), the prevalence was similar even after removing low-quality studies and studies that did not use standard criteria for diagnosis of high BP. All of these studies used a height-unadjusted standard cutoff BP of 140/90 mm Hg or the old height-adjusted guideline published in 2004.^23^ The high prevalence of high BP observed in the current CNNS, compared with earlier published Indian studies could partly be attributed to the age group studied, use of different cutoffs for diagnosing high BP, differences in the study design, and difference in the method of measuring BP.

Many earlier studies^19,20^ have either included children younger than 10 years or excluded children older than 16 years, whereas the CNNS included children only in the adolescent age group (aged 10-19 years). Some studies^11,21^ measured BP on more than 1 visit for confirmation. The diagnosis of high BP in the current study was based on the American Academy of Pediatrics guidelines published in 2017, whereas most other studies have used criteria previously developed in the fourth report from the National Institutes of Health’s National Heart, Lung, and Blood Institute in 2004 or other non-standard criteria.^14,23^ In studies that compared both criteria, the estimated prevalence of elevated BP was higher with the 2017 criteria compared with the 2004 criteria.^24^

Another differentiator is that although most earlier studies^19,22^ measured BP by the auscultatory method using a mercury sphygmomanometer, the CNNS used calibrated oscillometric automated devices.^13^ Higher SBP readings when using oscillometric devices have been noted in a systematic review and meta-analysis^25^ with a pooled effect estimate of 2.53 mm Hg (95% CI, 0.57-4.5 mm Hg) when compared with a standard mercury sphygmomanometer in children. Similarly, in a multicentric study^26^ conducted among children with chronic kidney disease, oscillometric SBP and DBP measurements were consistently higher than the readings obtained by auscultation (median elevations of 9 mm Hg for SBP and 6 mm Hg for DBP).

The prevalence of high BP noted here is similar to the high prevalence of hypertension reported in Indian adults, with almost 33% of adults observed to have high BP in 2 national-level surveys (with 180 335 and 1 320 555 participants, respectively).^27,28^ The current findings indicate that monitoring for high BP should begin in early adolescence, because reports on BP trajectory curves from childhood to adulthood demonstrate that BP values in the higher part of the BP distribution in childhood progress to hypertension in young adulthood.^29,30^

The prevalence of high BP noted here is also much greater than similar surveys conducted in other countries with national, multistage sampling designs, which also used the 2017 criteria to classify BP. One of the oldest and longest surveys available is the US National Health and Nutrition Examination Survey (NHANES), which has been conducted in 2-year cycles since 1999.^31^ The prevalence of high BP in 2015-2018 was 4.6% (95% CI, 3.4%-5.9%) among US children aged 8 to 12 years and 3.7% (95% CI, 2.6%-4.7%) among children aged 13 to 17 years.^32^ In the China Health and Nutrition Survey from 2015, the prevalence of high BP was higher than the NHANES prevalence but lower compared with the current Indian CNNS (18.2% [95% CI, 15.6%-21.0%] among children aged 7-12 years and 22.1% [95% CI, 17.6%-27.5%] among children aged 13-17 years).^33^ The National Health Survey of Pakistan estimated that nearly 18.9% of the people in Pakistan aged 15 years or older had hypertension.^34^ The Canadian Health Measures Survey of 2007 to 2015 reported an overall prevalence of high BP of 5.8% (95% CI, 5.0%-6.6%) in children aged 6 to 17 years.^35^ More children with high BP were observed in the 8- to 12-year group compared with the 13- to 19-year group, similar to that observed in the NHANES. In the Canadian survey, children with high BP were younger (mean age, 10.5 years) than those with normal BP (mean age, 12.3 years).

The similar prevalence of high BP in rural and urban children runs contrary to some earlier data, which indicated that the prevalence of high BP in children residing in urban regions was almost twice that observed in children from rural regions.^36,37^ The higher prevalence of high BP in rural areas, which was observed in the current CNNS, may be attributable to their lower socioeconomic status as well as the rapid urbanization of rural India, resulting in altered obesogenic dietary habits and lower level of physical activity. Indeed, adolescents in the lower age group belonging to richer category of wealth had a lower prevalence of high BP (PR, 0.82; 95% CI, 0.70-0.96) compared with children from the poorest category. Consistent with prior studies,^38,39^ the CNNS data also demonstrated an association between overweight or obesity and a higher prevalence of high BP, more so in younger adolescents. Prior studies^40,41^ have also documented the effect of overweight or obesity on SBP and DBP in children as young as 2 to 5 years and indicate a strong association between BP and BMI in early life.

With respect to the double burden of malnutrition, a previous analysis^11^ of the CNNS data showed the presence of metabolic obesity biomarkers in more than 50% of anthropometrically undernourished and normal-weight Indian children and adolescents. However, that analysis did not include BP. Our analysis has shown a synergistic effect of stunting and overweight toward high BP in younger children, with a more than 50% increased prevalence of high BP in children with stunting who also have overweight or obesity compared with children with stunting who have a normal weight appropriate for their height.

High BP was found to coexist with other cardiovascular disease risk factors. As in adults, high BP in children has also been shown to coexist with other cardiovascular diseases risk factors, particularly overweight and obesity.^42,43,44^

### Strengths and Limitations

The strengths of these findings are that for the first time in India a national population-based, quality-controlled survey is available in which BP was recorded in a large number of adolescents (aged 10-19 years) along with measures of other exposures. We also provide insight into the relatively unexplored association of BP with stunting and double burden of malnutrition. However, the study has some limitations. The reported estimates for high BP are based on 1 visit, using an undocumented oscillometric device for BP measurement. Because this is a cross-sectional survey, causality cannot be inferred. The prevalence of overweight and obesity, which is positively associated with high BP, was 3% higher in the analyzed sample compared with those not considered for analysis because of the missing BP measurements, which could have slightly overestimated the prevalence. Data on some potential factors associated with hypertension, such as prematurity, birth weight, nutrition-related factors, and physical activity, were not available for exploration. The details of exclusion from sampling for BP measurements are not available from the original study. In the absence of reference nomograms of BP in healthy, normal-weight Indian children, the 2017 guideline of the American Academy of Pediatrics was used, the effect of which on estimates of high BP prevalence is unknown.

## Conclusions

In this nationally representative survey of adolescents in India, we found an unexpectedly large burden of high BP in Indian children along with clustering of cardiovascular disease risk factors. Given that children with high BP are likely to become adults with high BP, with all the attendant sequelae, these findings warrant an urgent focus on screening programs and suitable public health policies to mitigate this malady.

## Notes

Supplement. **eTable 1. **American Academy of Pediatrics 2017 Guidelines Used for the Classification of BP as Elevated, Stage 1 or Stage 2 Hypertension **eTable 2. **Child Characteristics by BP Data Availability **eTable 3. **Prevalence of High BP in BMI Categories **eTable 4.** Association of Overweight/Obesity With High BP in Height Categories **eTable 5.** Co-occurrence of Multiple Cardiometabolic Risk Factors and Its Association With High BP **eFigure.** Distribution of Systolic (Panel A) and Diastolic (Panel B) Blood Pressure by Age and Sex
