# Supplementary material for: Prevalence of and Factors Associated With High Blood Pressure Among Adolescents in India
Source: JAMA Netw Open. 2022 Oct 31;5(10):e2239282. doi: 10.1001/jamanetworkopen.2022.39282 (PMC9623439; doi:10.1001/jamanetworkopen.2022.39282)
Supplement: Supplement. — eTable 1. American Academy of Pediatrics 2017 Guidelines Used for the Classification of BP as Elevated, Stage 1 or Stage 2 Hypertension eTable 2. Child Characteristics by BP Data Availability eTable 3. Prevalence of High BP in BMI Categories eTable 4. Association of Overweight/Obesity With High BP in Height Categories eTable 5. Co-occurrence of Multiple Cardiometabolic Risk Factors and Its Association With High BP eFigure. Distribution of Systolic (Panel A) and Diastolic (Panel B) Blood Pressure by Age and Sex [file jamanetwopen-e2239282-s001.pdf]

Supplementary Online Content

Vasudevan A, Thomas T, Kurpad A, Sachdev HS. Prevalence of and factors associated with high blood pressure among adolescents in India. *JAMA Netw Open*. 2022;5(10):e2239282. doi:10.1001/jamanetworkopen.2022.39282

eTable 1. American Academy of Pediatrics 2017 Guidelines Used for the Classification of BP as Elevated, Stage 1 or Stage 2 Hypertension
eTable 2. Child Characteristics by BP Data Availability
eTable 3. Prevalence of High BP in BMI Categories
eTable 4. Association of Overweight/Obesity With High BP in Height Categories
eTable 5. Co-occurrence of Multiple Cardiometabolic Risk Factors and Its Association With High BP
eFigure. Distribution of Systolic (Panel A) and Diastolic (Panel B) Blood Pressure by Age and Sex

This supplementary material has been provided by the authors to give readers additional information about their work.

© 2022 Vasudevan A et al. *JAMA Network Open.*

eTable 1. American Academy of Pediatrics 2017 guidelines used for the classification of BP as elevated, Stage1 or Stage 2 hypertension (Reproduced from Ref 13)

	**Children aged 10-12 years**	**Children aged ≥13 to 19 years**	**Referred to as high BP in the paper**
**Normal BP**	<90th percentile	<120/80 mmHg	No
**Elevated BP**	≥90th percentile to 95th percentile or 120/80 mmHg to <95th percentile (whichever is lower)	120/<80 to 129/<80 mmHg	
**Stage 1 hypertension**	≥95th percentile to <95th percentile +12 mmHg, or 130/80 to 139/89 mmHg (whichever is lower)	130/80 to 139/89 mmHg	Yes
**Stage 2 hypertension**	≥95th percentile + 12 mmHg, or ≥140/90 mmHg (whichever is lower)	≥140/90 mmHg	

© 2022 Vasudevan A et al. *JAMA Network Open.*

eTable 2. Child characteristics by BP data availability

	BP data not available (n=4510)	BP data available (n=11672)
Age group		
10-12y	32.6	32.7
	(30.4,34.9)	(30.8,34.7)
Area		
Rural	76.8	73.1
	(71.5,81.4)	(69.3,76.7)
Sex		
Male	47.5	51.9
	(44.8,50.2)	(50.0,53.7)
Wealth Index		
Poorest	24.2	15.4
	(20.6,28.2)	(12.8,18.5)
Poor	21.4	20.6
	(18.9,24.1)	(18.6,22.8)
Middle	19.3	21.3
	(17.1,21.8)	(19.6,23.1)
Rich	18.6	22.1
	(16.1,21.5)	(20.3,24.0)
Richest	16.4	20.5
	(14.1,19.0)	(18.2,23.1)
BMI Categories		
Overweight/Obese	2.9	5.8
	(2.2,3.8)	(5.0,6.7)

Values are % (95% Confidence Interval)

© 2022 Vasudevan A et al. *JAMA Network Open.*

e Table 3 Prevalence of high BP in BMI categories

BMI Categories	Prevalence of high BP* % (95% Confidence Interval)	
	Age 10-12y	Age ≥13 y
Underweight	32.2% (95% CI: 27-38)	21.5% (95% CI: 17-26)
Normal	35.1% (95% CI: 31-40)	24.9% (95% CI: 22-29)
Overweight	31.6% (95% CI: 31-44)	34.8% (95% CI: 28-42)
Obese	49.6% (95% CI: 31-68)	35.4% (95% CI: 22-51)

Underweight- WHO BMI z score<-2, Normal- WHO BMI z score≥-2 & <1, Overweight- WHO BMI z score≥1& <2, Obese- WHO BMI z score≥2

** survey weighted prevalence estimates with national sampling weights*

© 2022 Vasudevan A et al. *JAMA Network Open.*

eTable 4. Association of overweight/obesity with high BP in height categories

Age group	Category	PR (95% Confidence Interval)
10-12 y	Non-Stunted (n=2984)	1.17(1.02,1.35)
	Stunted (n=748)	1.52(1.18,1.96)
≥13 y	Non-Stunted (n=5244)	1.32(1.15,1.50)
	Stunted (n=1844)	1.35(1.04,1.77)

PR – Adjusted prevalence ratio with 95% Confidence interval, area, sex, wealth index, BMI category considered in the model

© 2022 Vasudevan A et al. *JAMA Network Open.*

eTable 5. Co-occurrence of multiple cardiometabolic risk factors and its association with high BP

Number of cardiometabolic risk factors the child has	Prevalence of high BP, % (95% Confidence Interval)	Unadjusted PR (95% Confidence Interval) of high BP	Adjusted PR* (95% Confidence Interval) of high BP
Age <13 y			
None(n=929)	33.0(27.0,49.7)	Ref.	Ref.
One(n=1066)	36.0(29.5,42.9)	1.24(1.11,1.38)	1.24(1.11,1.38)
Two(n=474)	48.2(39.1,57.5)	1.46(1.28,1.66)	1.48(1.30,1.68)
Three or Four (n=125)	43.1(25.8,62.2)	1.36(1.10,1.68)	1.41(1.14,1.75)
Age≥13 y			
None(n=2023)	19.8(17.3,24.4)	Ref.	Ref.
One(n=1850)	20.0(21.8,30.6)	1.09(0.97,1.22)	1.10(0.98,1.24)
Two(n=837)	25.6(25.7,64.6)	1.38(1.21,1.57)	1.39(1.22,1.58)
Three or Four (n=248)	53.3(42.7,98.5)	1.60(1.33,1.93)	1.62(1.35,1.95)

PR-Prevalence Ratio

** Adjusted prevalence ratio with 95% Confidence interval, age, area, sex, wealth index, BMI category considered in the model*

© 2022 Vasudevan A et al. *JAMA Network Open.*

eFigure. Distribution of systolic (Panel A) and diastolic (Panel B) Blood Pressure by age and sex.

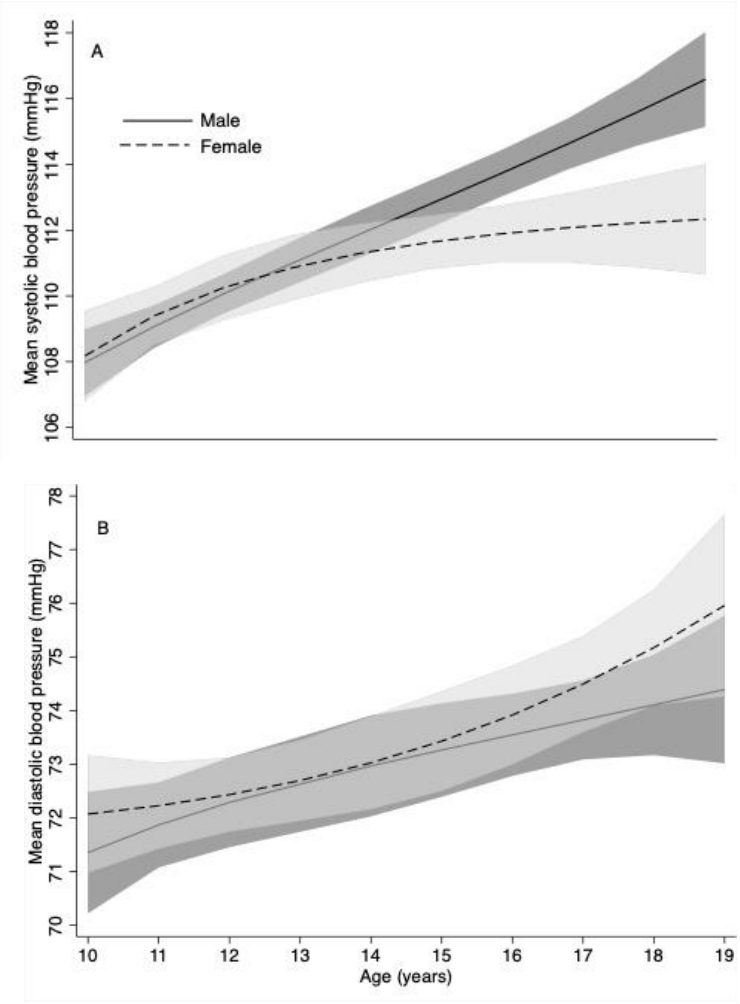

Distribution of systolic (A) and diastolic (B) Blood Pressure by age and sex. Y axis is mean blood pressure (mmHg) and X axis is age of the child (years). Solid line represents male children and dashed line represent female children. The shaded area is the 95% Confidence interval for the fit line.

© 2022 Vasudevan A et al. *JAMA Network Open.*
